# The Impact of Inpatient Telemedicine on Personal Protective Equipment Savings During the COVID-19 Pandemic: Cross-sectional Study

**DOI:** 10.2196/28845

**Published:** 2021-05-19

**Authors:** Reem Halabi, Geoffrey Smith, Marc Sylwestrzak, Brian Clay, Christopher A Longhurst, Lina Lander

**Affiliations:** 1 School of Medicine University of California, San Diego San Diego, CA United States; 2 Information Services University of California San Diego Health San Diego, CA United States; 3 Health Sciences Department of Biomedical Informatics University of California, San Diego San Diego, CA United States; 4 Department of Medicine University of California, San Diego San Diego, CA United States; 5 Department of Pediatrics University of California, San Diego San Diego, CA United States; 6 Department of Family Medicine University of California, San Diego La Jolla, CA United States

**Keywords:** inpatient telemedicine, bedside iPad, video visits, personal protective equipment, COVID-19, virtual visits, pandemic, telehealth, telemedicine, digital health

## Abstract

With the emergence of the COVID-19 pandemic and shortage of adequate personal protective equipment (PPE), hospitals implemented inpatient telemedicine measures to ensure operational readiness and a safe working environment for clinicians. The utility and sustainability of inpatient telemedicine initiatives need to be evaluated as the number of COVID-19 inpatients is expected to continue declining. In this viewpoint, we describe the use of a rapidly deployed inpatient telemedicine workflow at a large academic medical center and discuss the potential impact on PPE savings. In early 2020, videoconferencing software was installed on patient bedside iPads at two academic medical center teaching hospitals. An internal website allowed providers to initiate video calls with patients in any patient room with an activated iPad, including both COVID-19 and non–COVID-19 patients. Patients were encouraged to use telemedicine technology to connect with loved ones via native apps or videoconferencing software. We evaluated the use of telemedicine technology on patients’ bedside iPads by monitoring traffic to the internal website. Between May 2020 and March 2021, there were a total of 1240 active users of the Video Visits website (mean 112.7, SD 49.0 connection events per month). Of these, 133 (10.7%) connections were made. Patients initiated 63 (47.4%) video calls with family or friends and sent 37 (27.8%) emails with videoconference connection instructions. Providers initiated a total of 33 (24.8%) video calls with the majority of calls initiated in August (n=22, 67%). There was a low level of adoption of inpatient telemedicine capability by providers and patients. With sufficient availability of PPE, inpatient providers did not find a frequent need to use the bedside telemedicine technology, despite a high census of patients with COVID-19. Compared to providers, patients used videoconferencing capabilities more frequently in September and October 2020. We did not find savings of PPE associated with the use of inpatient telemedicine.

## Expansion of Telemedicine and Patient iPad Program

In response to the COVID-19 pandemic, health care organizations rapidly implemented telemedicine protocols to protect health care workers and patients [1-3]. The anticipated surge in patients and shortage in personal protective equipment (PPE) were key drivers for the implementation of virtual visits and medical screening exams in the inpatient settings and emergency departments [4]. Some authors referred to the inpatient telemedicine as “electronic PPE” [4], and a few studies saw a decrease in patient contact, resulting in PPE savings [5,6]. One study among emergency department patients found telemedicine to be associated with a significant reduction in PPE use, without anticipated increase in anxiety and dissatisfaction among patients [6]. Overcoming operational barriers such as privacy, device availability, and functionality may enable organizations to conserve PPE while providing excellent patient care [7]. Although a few studies described the deployment of hospital tablets for inpatient telemedicine, the extent of long-term continuous use of this technology has not been extensively evaluated [8,9].

University of California (UC) San Diego Health is an academic medical center with multiple outpatient ambulatory sites and two hospital campuses with 808 licensed inpatient beds. Bedside iPads were implemented at one affiliated teaching hospital in 2016 followed by expansion in 2019 to include the entire inpatient population [10]. The original intent of the bedside iPads was to allow patients access to their inpatient electronic health record portal (Epic MyChart Bedside), hospitality (ordering food), comfort (controlling ambient lights and room temperature), and entertainment (streaming services) [10]. As the COVID-19 pandemic evolved and more patients required isolation precautions, hospitals limited visitations by family members. Bedside iPads, therefore, appeared to be a natural solution to connect patients with their loved ones and to conserve PPE by allowing clinicians to interact with patients remotely. The objective of this viewpoint is to describe the use of a rapidly deployed inpatient telemedicine workflow at a large academic medical center and discuss technology’s potential impact on PPE use.

## Inpatient Telemedicine Process Implementation

**Figure 1:**
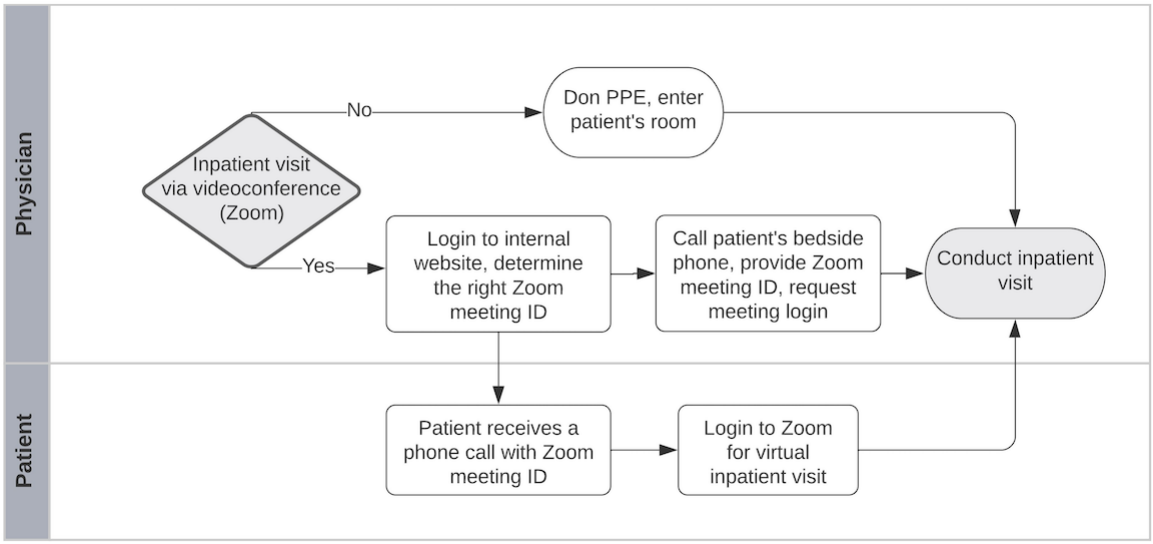
Process flow for inpatient video call at an academic medical center in San Diego, CA. PPE: personal protective equipment.

A videoconferencing tool (Zoom, Zoom Video Communications) was implemented with health care security protocols in 2019 prior to the COVID-19 pandemic. The native Zoom app was installed on all the bedside iPads to allow patients to connect with their families using video calls. Inpatient providers could also initiate Zoom calls with patients. At the beginning of the pandemic, providers could call the patient’s bedside telephone to provide them with a meeting ID to join a Zoom videoconference. The patient would then join the meeting initiated by the provider (Figure 1).

Although this process was adopted by many inpatient services, providers reported difficulties with retrieving telephone numbers for inpatient rooms. As a result, an internal website was created to display all available active patient iPads. From this website, providers could initiate a video call with a patient using a “Meet Now” button. The bedside iPad would then produce an audible ping alerting the patient of an incoming Zoom call. Upon discharge, the bedside iPad and Zoom ID were reset to ensure patient data privacy.

To evaluate the use of video calls in inpatient services and estimate the potential impact on PPE savings, we obtained data from the internal web server logs and Google analytics. Data from video calls to patients hospitalized for COVID-19 and other conditions were included. All available traffic data from the internal website were collected using internal servers between May and June 2020, before switching to using Google Analytics in July 2020.

We used Google Analytics to track user engagement events on patients’ iPads. Engagement events included clicking on the Start Meeting, Send Email, and Meet Now links. The Start Meeting button allowed a patient to initiate a video call with their family members or a provider and was used as an indicator for a patient-initiated video call. The Send Email button allowed a patient to email video call instructions to their family members and was used as another indicator of a patient-initiated call. The Meet Now button allowed a physician to initiate a video call with a patient and was used as an indicator for provider-initiated video calls.

It was not technically feasible to track the volume of telephone calls from providers to patient rooms to determine the frequency of phone or video visits during the initial stage of Zoom implementation. It was also not feasible to track Zoom meetings conducted through the native Zoom app due to UC San Diego Health cybersecurity protocols.

We did not collect any patient identifying information and followed all data safety protocols. We relied on server and Google analytics data to determine whether patients used the bedside iPads to participate in a video call or to send an email, not the duration or content of such engagements.

## Providers and Patients Underused Inpatient Telemedicine

**Figure 2:**
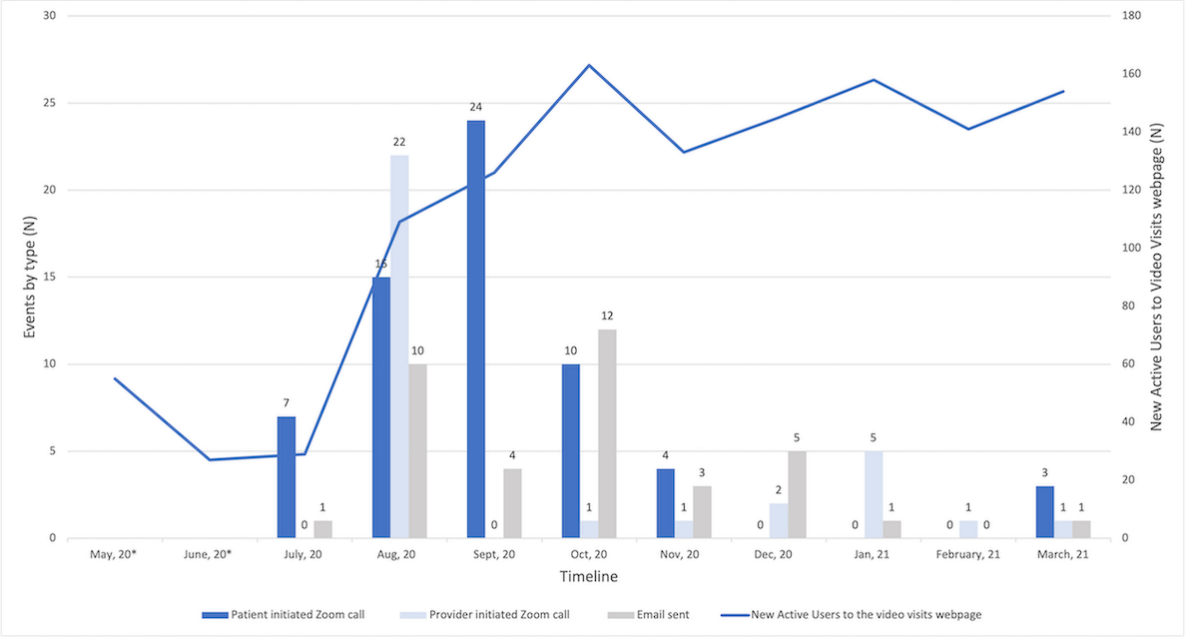
Users of the Video Visits webpage and engagement events (patient- and provider-initiated calls, emails), May 2020-March 2021, San Diego, CA. *The number of users of the Video Visits website was determined from the internal server data for May-June 2020 and from Google Analytics for July 2020-March 2021.

The number of active users on the Video Visits website was determined from the internal server data for May and June 2020, and from Google Analytics for July 2020 to March 2021. There were a total of 1240 events collected from both data sources. On average, between May 2020 and March 2021, there were 112.73 (SD 49) connection events per month (Figure 2). As expected, use increased between July (n=29) and March (n=154). The lowest use was recorded in June (n=27) and the highest in October (n=163), followed by January 2021 with 158 users (Figure 2, line).

Although the number of visitors to the Video Visits site was low, the actual engagement with the system was even lower with 133 (10.7%) connections out of the total 1240 events. Of these 133 connections, patients initiated 63 (47.4%) video calls with family or friends and sent 37 (27.8%) emails with connection instructions. The highest number of calls occurred in September 2020 (n=24, 38%), and the largest number of emails were sent in October (n=12, 32%). Provider use was even lower, with providers initiating a total of 33 (52%) video calls with patients. The majority of calls were initiated in August (n=22, 67%) perhaps due to an internal awareness campaign of the available capability. Because the servers filter out automatic page refreshes, these numbers may underestimate the total number of video calls.

## Inpatient Telemedicine Could Be Used to Conserve PPE

Although we implemented convenient workflows for clinicians to contact patients virtually to reduce the risk for infection, save PPE, and potentially save time to don and doff PPE, we saw minimal adoption of inpatient telemedicine. There appeared to be some seasonal variation in use, and during our study, patients initiated higher number of calls than providers (63 vs 33). The majority of our provider calls occurred in 1 month (August 2020) possibly due to an internal awareness campaign in response to a COVID-19 wave occurring at the time. Use declined to 0 to 5 calls in subsequent months, however. Our results are different from those of previous studies that found high inpatient telemedicine use and subsequent savings of PPE [4-6,8].

No technical issues with iPads or Zoom were reported, yet the vast majority of providers saw patients in person. Inpatient physicians may prefer to see patients in person, regardless of availability and convenience of technology or potential saving of PPE. Fortunately, our academic medical center has not experienced the PPE shortages faced by our colleagues in other parts of the country [11]. As such, there was never a significant impetus to conserve PPE using all available means including inpatient telemedicine capability.

Our study had several limitations including small sample size and limited scope. Although most clinicians were comfortable with Zoom, it is possible that some were not, which would reduce technology use for inpatient visits. Patients have various comfort levels with technology, which could reduce the potential usability of inpatient telemedicine. In addition, we did not evaluate the utility of inpatient telemedicine for other health care providers such as nurses and technicians.

With the COVID-19 pandemic, telemedicine capacity was rapidly implemented in ambulatory settings [1-3]. Emergency departments also found multiple use cases for telemedicine including patient triage and expedited care for stable patients, reduced potential provider exposure to COVID-19, decreased use of PPE, reducing patient isolation, and allowing quarantined physicians to continue practicing, among others [4-6,12,13].

The utility of telemedicine in inpatient settings and its potential impact on PPE savings, however, remains to be addressed. A few studies that found inpatient telemedicine to be useful for patient care and PPE savings also reported operational barriers such as privacy and security, and limited functionality and device availability [7-9]. The availability of the technology by itself was inadequate to encourage use while PPE supplies were sufficient, and additional studies are needed to evaluate the utility of inpatient telemedicine for health care providers.

## Conclusions

Similar to outpatient clinics and emergency departments, telemedicine capability could have a positive impact in inpatient settings including PPE savings. Our study found low adoption levels of inpatient telemedicine among patients and providers. Despite a high census of patients with COVID-19, our providers saw patients in person rather than relying on telemedicine. Patients’ use of bedside iPads appeared to be limited for telemedicine and should continue to be evaluated to improve patient experience.

Increasing awareness of telemedicine in inpatient settings for providers and patients could help increase use for patient care. Going forward, implementation of telemedicine across all levels of care may help reduce potential provider exposure to COVID-19 and increase provider capacity.

## Abbreviations

PPE: personal protective equipment
UC: University of California
